# The Application of the Grey Disaster Model to Forecast Epidemic Peaks of Typhoid and Paratyphoid Fever in China

**DOI:** 10.1371/journal.pone.0060601

**Published:** 2013-04-02

**Authors:** Xuejun Shen, Limin Ou, Xiaojun Chen, Xin Zhang, Xuerui Tan

**Affiliations:** 1 Department of Cardiology, The First Affiliated Hospital of Shantou University Medical College, Shantou, Guangdong Province, China; 2 Department of Neurology, The First Affiliated Hospital of Shantou University Medical College, Shantou, Guangdong Province, China; 3 Laboratory of Molecular Cardiology, The First Affiliated Hospital of Shantou University Medical College, Shantou, Guangdong Province, China; 4 Shantou University Medical College, Shantou, Guangdong Province, China; Indian Institute of Science, India

## Abstract

**Objective:**

The objectives of this study were to forecast epidemic peaks of typhoid and paratyphoid fever in China using the grey disaster model, to evaluate its feasibility of predicting the epidemic tendency of notifiable diseases.

**Methods:**

According to epidemiological features, the GM(1,1) model and DGM model were used to build the grey disaster model based on the incidence data of typhoid and paratyphoid fever collected from the China Health Statistical Yearbook. Model fitting accuracy test was used to evaluate the performance of these two models. Then, the next catastrophe date was predicted by the better model.

**Results:**

The simulation results showed that DGM model was better than GM(1,1) model in our data set. Using the DGM model, we predicted the next epidemic peak time will occur between 2023 to 2025.

**Conclusion:**

The grey disaster model can predict the typhoid and paratyphoid fever epidemic time precisely, which may provide valuable information for disease prevention and control.

## Introduction

Typhoid fever and paratyphoid fever are systemic infections caused by *Salmonella enterica*, including *S enterica* serotype Typhi and serotypes Paratyphi A, B, and C [1], [2]. As a faecal–oral transmissible disease, in developing countries, it is associated with economic backwardness, poor sanitation, and unsafe food or water, whereas in developed countries, incidence is mainly caused by returned travelers to developing countries [3]. Typhoid and paratyphoid fever have been effectively controlled in Europe and North America, in spite of that, the incidence remains high in some developing countries in Asia, Africa, and South America [4]. It is still an important public health problem [5], to which much attention has been paid. Based on the previous publications in recent years, the key points of research still focus on epidemiology [6]–[8], pathogenesis [9], [10], diagnosis [11]–[13], treatment [14], [15] and control [16], [17]. Incidence of a disease can be used for predicting the number of new cases in the years to come [18], which will provide decision references for planning prevention and control. Herein, time series analysis [19], [20], D-R model, GM(1,1) model [21], [22], Markov chain prediction model [23] and multi-variate linear regression [24] have been used to predict future trends in some infectious diseases. However, these published forecasting methods mostly aim at the incidence, prevalence, or mortality rate (or the number of people) of a disease, rather than the time when an epidemic peak may occur.

The grey systems theory, established by Julong Deng in 1982, is a new methodology that chiefly includes the theory of grey system analysis, modeling, prediction, decision-making and control [25]. It focuses on uncertainty problems with small sample, discrete data and incomplete information that are difficult for probability and fuzzy mathematics to handle. Grey prediction is an important embranchment of grey systems theory, which makes scientific, quantitative forecasts about the future states of grey systems. Based on their functions and characteristics, grey predictions can be grouped into sequence predictions, disaster predictions, seasonal disaster predictions, stock-market-like predictions, system predictions, etc. And they are realized through establishing grey models for relevant sequences. Among them, grey disaster prediction is essentially the prediction for time distribution of abnormal values, and aims at forecasting the exact moments of the forthcoming catastrophe to help relevant parties to prepare ahead of time for the worst condition.

GM(1,1) model is the main and basic model of grey predictions, i.e. a single variable first order grey model, which is able to acquire high prediction accuracy despite requiring small sample size (but the sample size must be at least 4) [26], while some of the commonly used prediction methods such as regression analysis rely heavily on historical data and are required to satisfy a certain typical form of data distribution. Moreover, the method is straightforward because little computational effort is needed to constitute the prediction model. Although GM(1,1) model has been widely used, its predicting performance still could be improved sometimes. Discrete grey model (DGM) [27] is one of the most important methods to improve the precision of the model.

The grey prediction model has been widely applied in petroleum, geological engineering, medicine, industry, management, agriculture and other fields [28]–[33] and has obtained successful results. In the field of medicine and public health, the occurrence, development, and prevalence of a disease is affected by many uncertain factors, can be regarded as a dynamic changing grey system, and is an appropriate application for grey prediction. So far, grey prediction has been widely used in morbidity and mortality prediction of a variety of infectious disease epidemics [21], [34], parasitic disease, and malignant tumor.

As one of the category B notifiable diseases in China, although typhoid and paratyphoid fever has been effectively controlled nationwide, the prevalence in some regions is still high [35]. Taking into account the characteristics of infectious diseases, we used the grey disaster model to analyze typhoid and paratyphoid fever incidence data from 1975 to 2010 and then compared performance of two methods: the GM(1,1) model and the DGM model. Finally, the better one was used to extrapolate future trends. We propose that the grey disaster model is able to define the time distribution of typhoid and paratyphoid fever in China and the result may provide useful references for controlled application.

## Materials and Methods

### Data sources

The typhoid and paratyphoid incidence data was collected from the China Health Statistical Yearbook of 2011 [36], published by the Ministry of Health of the People's Republic of China, which was open government statistics data.

### Methods

The grey disaster prediction modeling process and model test methods were established according to “Grey systems:theory and applications” [26] and “Elements of Grey Theory” [37]. Grey system theory modeling software 3.0 (GTMS3.0) was used for the statistical analysis.

#### A. **The principle of grey disaster prediction**

The basic idea of grey disaster prediction is to forecast the occurrence time of abnormal values. By specifying a threshold, abnormal values were picked out to form a subset series of the original series. Then a catastrophe date sequence is generated, based on which the grey prediction model is established to predict the exact moments of the forthcoming abnormal values. It is not for predicting the numerical value itself, but rather for predicting the time(s) of one or several abnormal values to occur.

#### B. Model construction

Time series data of typhoid and paratyphoid fever incidence year by year from 1975 to 2010 was considered as the original series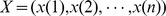. Incidence data significantly higher than the normal sporadic level was defined as an abnormal value and was picked out to form a subset series of the original series 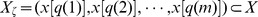, which was assumed to be a catastrophe sequence, and the corresponding catastrophe date sequence Q^(0)^ = (q(1), q(2), …, q(m)). The q(m) stood for the *m*th catastrophe time sequence value that typhoid and paratyphoid fever outbreak or epidemic during n years (1≦m≦n). Then, disaster prediction was made by establishing grey prediction models to forecast the catastrophe date in the future. In order to get better simulation accuracy, the GM(1,1) model and the DGM model were used for prediction in this study, and performances of these two models were compared by testing their simulation accuracy. According to the characteristics of our data, this study defined a disease epidemic time as the year when the incidence increased to a maximum value, and the value was more than 1.2 times the average of previous three years. That was taking the peak value of the incidence as an abnormal value.

The general procedure for a GM(1,1) model is derived as follows:

1. Original time sequence with n samples is expressed as 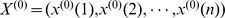, where 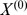 is a non-negative sequence and n is the sample size of the data.
2. First-order accumulative generation operation (1-AGO) is used to convert chaotic series 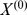 into monotonically increasing series: 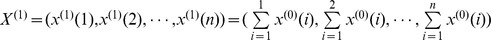.
3. The generated mean sequence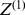 of 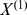 is defined as: 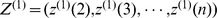, where 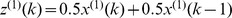
(k = 2, 3, …, n)is the mean value of adjacent data.
4. The least square estimate sequence of the grey difference equation of GM(1,1) is defined as:
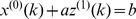


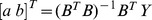
, where 
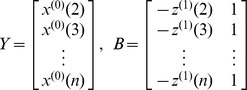

5. The whitenization equation is given by: 
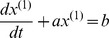

6. Solve the equation, the GM(1,1) forecasting model can be obtained: 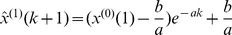

7. Predicted value of the primitive data at time point (k+1) is extracted: 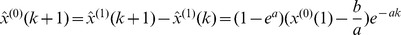

The establishment of DGM model:

1. The definition of DGM: The equation 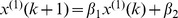 is called discrete grey model or discrete form of the grey model.

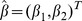
 is a sequence of parameters, and 
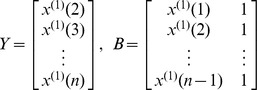
 then the least squares estimate sequence of the grey differential equation 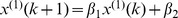 will satisfied to 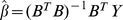

3. Set 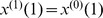, then recursive function is given by 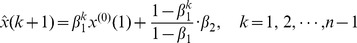 or 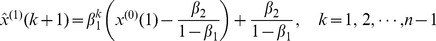

Then, the restored values of 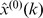 can be given by 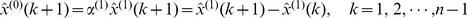


More details for GM(1,1) and DGM modeling steps can be seen in “Grey systems: theory and applications” [26].

#### C. Model fitting accuracy test

Model was tested to evaluate its appropriateness and effectiveness before application. The mean relative error, absolute degree of incidence, variance ratio and small error probability were used for testing. Simulation accuracy of each model was comprehensively evaluated according to Table 1.

**Table 1:** Commonly used scales of accuracy for model testing.

Accuracy scale	Mean relative error 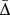	Degree of incidence ε	Variance ratio C	Small error probability p
1st level	≤0.01	≥0.90	≤0.35	≥0.95
2nd level	0.01∼0.05	0.80∼0.90	0.35∼0.50	0.80∼0.95
3rd level	0.05∼0.10	0.70∼0.80	0.50∼0.65	0.70∼0,80
4th level	0.10∼0.20	0.60∼0.70	0.65∼0.80	0.60∼0.70

The mean relative error 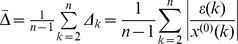, for a given *α*, when 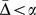 and 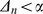 hold true, the prediction model is supposed to be error-satisfactory.

Let ε stand for the absolute degree of incidence between the raw data X^(0)^ and the simulated values. For a given ε_0_>0, if the absolute degree of incidence satisfies ε>ε_0_, then the simulation model is regarded to be incidence satisfactory.


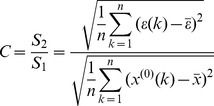
 is the variance ratio. For a given C_0_>0, if the ratio of variances C = S_2_/S_1_<C_0_, then the model is supposed to be variance ratio satisfactory.

If 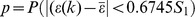 is taken as a small error probability and for a given *p*_0_>0, when *p*>*p*_0_, then the model is said to be small-error probability satisfactory.

#### D. Prediction of the Grey Model

The better model that has passed different tests can be used to make meaningful predictions of the epidemic peaks of typhoid and paratyphoid fever.

## Results

### Incidence of typhoid and paratyphoid fever from 1975 to 2010

**Figure 1:**
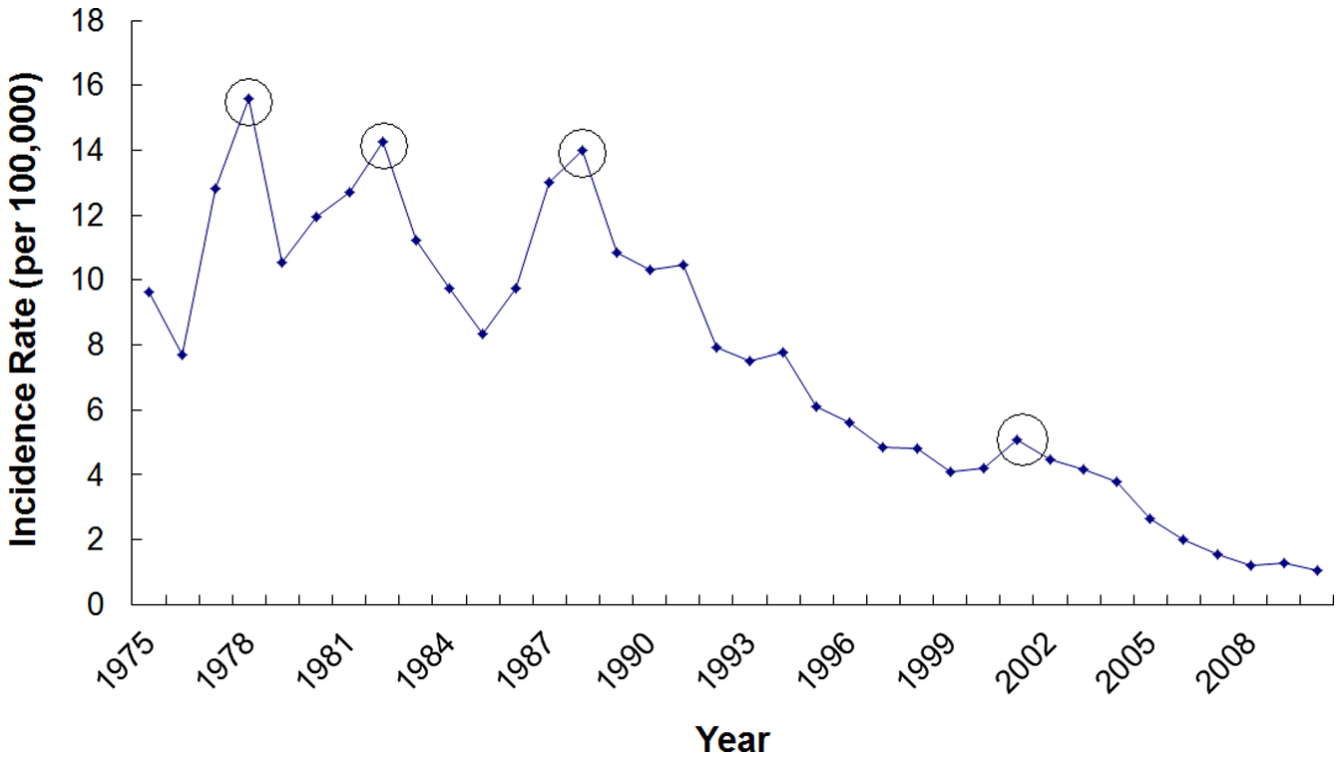
Incidence of typhoid and paratyphoid fever from 1975 to 2010. Sampling points in a circle were selected for analysis.

Incidence rates of typhoid and paratyphoid fever from 1975 to 2010 were shown in Figure 1 and Table 2. Over a 36-year period, incidence rates fluctuated, but the overall trend was downwards. In the chart (Figure 1), 4 peaks (years 1978, 1982, 1988, 2001) could be found, which might be considered as epidemic peaks. The first three peaks were more obvious than the fourth one and there was a 3-to-5-year interval between each of them. So this four data were initially selected to compare with the average incidence of previous three years. The results were as follows:

**Table 2:** Incidence of typhoid and paratyphoid fever from 1975 to 2010 (1/10^5^).

Year	Time series value	Incidence	Year	Time series value	Incidence
1975	1	9.61	1993	19	7.51
1976	2	7.68	1994	20	7.75
1977	3	12.82	1995	21	6.10
1978	4	15.58	1996	22	5.61
1979	5	10.53	1997	23	4.83
1980	6	11.94	1998	24	4.80
1981	7	12.72	1999	25	4.08
1982	8	14.25	2000	26	4.19
1983	9	11.24	2001	27	5.07
1984	10	9.75	2002	28	4.47
1985	11	8.35	2003	29	4.17
1986	12	9.76	2004	30	3.80
1987	13	13.02	2005	31	2.65
1988	14	14.01	2006	32	1.99
1989	15	10.83	2007	33	1.55
1990	16	10.32	2008	34	1.18
1991	17	10.45	2009	35	1.28
1992	18	7.91	2010	36	1.05

Note: underline data were abnormal values.

Incidence of year 1978 divided by that of the average of years 1975, 1976 and 1977: 15.58/((9.61+7.68+12.82)/3) = 1.55

In the same way, for year 1982, we got 14.25/((10.53+11.94+12.72)/3) = 1.21

For year 1988: 14.01/((8.35+9.76+13.02)/3) = 1.35

For year 2001: 5.07/((4.80+4.08+4.19)/3) = 1.16≈1.2

Obviously, incidence of years 1978, 1982 and 1988 were all more than 1.2 times of the average of the previous three years, and that of 2001 was 1.16 times, close to 1.2. For this set of data, we believed that the first three peaks could be defined as typhoid and paratyphoid fever epidemic peaks, and year 2001 could be considered as a small epidemic. Therefore, there were four outbreaks or epidemics in 1978, 1982, 1988 and 2001 during the past 36 years from 1975 to 2010. Thus, the corresponding catastrophe date sequence was Q(0) = (4, 8, 14, 27).

Modeling, model fitting accuracy test and comparison

The catastrophe date sequence was Q(0) = (4, 8, 14, 27), then GM(1,1) model and DGM model were constructed respectively based on this sequence.

Grey prediction equation constructed by GM(1,1) model was: 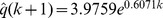


Grey prediction equation constructed by DGM model was: 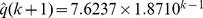


The simulative values and errors of GM(1,1) model and DGM model were shown in Table 3. Then the mean relative error, absolute degree of incidence, variance ratio and small error probability could be calculated to evaluate the model fitting accuracy (see Table 4).

**Table 3:** The simulative values and errors of GM(1,1) model and DGM model.

		GM(1,1) model		DGM model	
	Original value	Simulative value 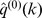	Relative error* (%)	Simulative value 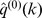	Relative error (%)
q^(0)^(1)	4	4.00	--	4.00	--
q^(0)^(2)	8	7.30	8.75	7.62△	4.75
q^(0)^(3)	14	13.39	4.36	14.26△	1.86
q^(0)^(4)	27	24.57	9.00	26.69△	1.15

Note: * Relative error  = 
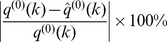

△ The simulative values of DGM model were close to the original values.

**Table 4:** Model fitting accuracy test results of GM(1,1) model and DGM model.

Model	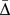 (accuracy scale)	ε (accuracy scale)	C (accuracy scale)	p (accuracy scale)
_GM(1,1)_	0.0737 (Third)	0.9514 (First)	0.1039 (First)	1 (First)
_DGM_	0.0259 (Second)	0.9948 (First)	0.0294 (First)	1 (First)

The mean relative error, absolute degree of incidence and variance ratio of DGM model were all superior to that of GM(1,1) model, which means that the simulation results of DGM model were better than that of GM(1,1) model.

### Predictions of the Grey Model

Since the simulation accuracy of GM(1,1) model and DGM model were all at the first level, they all could be used to make predictions. But according to the simulation results, the DGM model was finally chosen to make our predictions.

Substituting k = 4 into the model 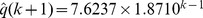, the calculated value was 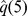 = 49.93.

The mean relative error of the DGM model derived from the results in Table 3 was: 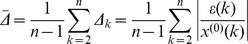 = (4.75%+1.86%+1.15%)/3 = 2.59%. In consideration of error rate, it would be more reasonable to describe the predicted value with interval numbers. Then the value was 49.93×(1±2.59%) = (48.64,51.22), which means the predicted interval was 49∼51.

This result indicated that the next epidemic peak should occur between the 49th∼51th year (taking 1975 as a base point 1), i.e. year 2023 to 2025.

## Discussion

For the grey disaster prediction model, a given upper/lower abnormal (or catastrophe) value is commonly determined based on the actual situation and individuals' experiences, and the subsequence is known as the upper/lower catastrophe sequence. However, the epidemic of infectious diseases has its own particularities. Epidemic is defined as the occurrence in a community or region of cases of an illness or other similar event clearly in excess of normal expectancy [38]. The characteristics of the illness, the area, the season and certain historical circumstances all have to be taken into account, so the criteria must be adapted to different periods. The use of the historical data by the national outbreak automatic detection and response system of China supports this concept, too. The system doesn't take advantage of all the historical data to enable early warning for the 19 kinds of infectious diseases, such as typhoid and paratyphoid fever. Instead, it performs the mobile percentile method, which uses the referential historic data of the last 3∼5 years only as baseline data, to dynamically correct the early-warning threshold value [39]. It has been reported that the incidence of 18 kinds of infectious diseases from 1970 to 2007, including typhoid and paratyphoid fever, had decreased from 4000∼4340/10^5^ to 120∼250/10^5^ people [40]. Since the establishment of the People's Republic of China, along with the development of the economy, the improvement of sanitary conditions and the popularity of the centralized water supply, great achievements have been made in the prevention and control of typhoid and paratyphoid fever. Simultaneously, waterborne typhoid outbreak has been significantly reduced. During 1995 to 2004, the number of cases of morbidity and mortality of typhoid and paratyphoid fever has decreased annually. Some poverty-stricken areas are still at high risk because of uneven economic development, and a large number of typhoid carriers remain after a previous outbreak that can lead to a fresh outbreak [35]. Therefore, using a single threshold value to determine the abnormal value of different historical periods is not quite reasonable. The prevalence of disease is affected by not only its inherent periodic variation or long-term trends, but also effective prevention and control measures by the government. The epidemic trend of most of the diseases experiences a rise at first, then a decline, and the epidemic curve is displayed as a multi-peak curve with varying heights. Prior to 1990, peaks were obviously higher than in the years after 1990. China began the implementation of the socialist market economy in the 1990s. We speculated that social civilization and progress of health conditions caused by this significant economic policy was an epochal shift for infectious diseases too, which not only changed the original trends, but also decreased the peak value. Taking all these aspects and the data features into consideration, we took the years, when incidence of typhoid and paratyphoid fever rose to the highest and was 1.2 times higher than the average of previous three years, as the abnormal points in the application of the grey disaster prediction model. The peak value of year 2001 was not as obvious as the other three, but still could be considered as a small epidemic during a different historic period. The emergence of the peak might be because of an increase in sporadic cases, or disease outbreak in some regions. Close attention should be paid to both situations, especially the second one.

In this study, the GM(1,1) model and DGM model were used to construct the grey disaster model. The simulation results showed that the DGM model had better performance. Then we chose the DGM model to make the prediction and found that the fifth epidemic peak time will occur between 2023 to 2025.

We used the grey disaster prediction model to analyze the incidence data of typhoid and paratyphoid fever based on its special characteristics in China, and demonstrated that application of grey prediction resulted in satisfactory accuracy. This indicates that the grey disaster forecast model enables prediction of epidemic peaks of typhoid and paratyphoid fever. Thus, grey prediction can be used to provide a foundation for planning and management decisions to prevent and control this disease.

Although the grey disaster prediction model has yielded good results, it may not be suitable for other specific situations. The grey model is more accurate when modeling for short- term forecasting, but for long-term forecasting, the accuracy may be degraded [22]. In addition, the epidemic of disease is inevitably affected by many factors, including external factors and health factors that can change disease progression. By reviewing the recently published literature for reports that evaluated methods for the detection of infectious disease outbreaks in public health surveillance data, Watkins *et al*. categorized evaluation methods into four main methods: the descriptive, derived, epidemiological and simulation approaches, and concluded that no single approach could fulfill all evaluation requirements [41]. So, the appropriate model should be selected through comprehensive consideration and conscientious analysis according to the actual circumstance, and be promptly adjusted in relation to specific conditions in the community, changes in the pattern of life and the natural history of disease. Only after that can we take full advantage of prediction models to optimize long-term benefits.
