# Correction: The Application of the Grey Disaster Model to Forecast Epidemic Peaks of Typhoid and Paratyphoid Fever in China

**DOI:** 10.1371/annotation/1ccac7c6-e139-404a-bed6-584666913dbc

**Published:** 2013-08-21

**Authors:** Xuejun Shen, Limin Ou, Xiaojun Chen, Xin Zhang, Xuerui Tan

There were multiple typographical errors in the first sentence of the Funding Statement. The first sentence of the Funding Statement should read: "This work was supported by the National Natural Science Foundation of China (No. 81172776, www.nsfc.gov.cn) and Project for High-level Talents in Guangdong Higher Education (www.gdhed.edu.cn)." 

